# Effect of Transport Distance and Season on Some Defects of Fresh Hams Destined for DPO Production

**DOI:** 10.3390/ani4030524

**Published:** 2014-08-29

**Authors:** Agnese Arduini, Veronica Redaelli, Fabio Luzi, Stefania Dall’Olio, Vincenzo Pace, Leonardo Nanni Costa

**Affiliations:** 1 Department of Agricultural and Food Science, School of Agriculture and Veterinary Medicine, University of Bologna, Via Fanin 50, 40127 Bologna, Italy; E-Mails: veronica.redaelli@unibo.it (V.R.); stefania.dallolio@unibo.it (S.D.); leonardo.nannicosta@unibo.it (L.N.C.); 2 Department of Health, Animal Science and Food Safety, Faculty of Veterinary Science, University of Milan, Via Celoria 10, 20133 Milan, Italy; E-Mail: fabio.luzi@unimi.it; 3 OPAS, Pig Farmer Association, Strada Ghisiolo 57, 46030 San Giorgio, Mantua, Italy; E-Mail: vincenzo.opas@coopgsp.it

**Keywords:** pigs, transport, distance, season, DPO Parma ham, pre-slaughter handling, defect

## Abstract

**Simple Summary:**

Transport to the slaughterhouse is a stressful event for pigs. Travel duration and conditions can negatively affect animal welfare and carcass quality. Some defects in fresh hams are strictly connected to pre-slaughter transportation. Journeys with short (<37 km) and long (>170 km) distances may increase damage in fresh hams and decrease Denomination Protected of Origin (DPO) Parma dry-cured ham production.

**Abstract:**

Pre-slaughter handling is related to defects in fresh hams that result in exclusion from the DPO Parma chain, including hematomas, lacerations, microhaemorrhages and veining. To determine the effects of transport conditions on hams, we collected data on defects in 901,990 trimmed fresh hams from heavy pigs provided by 3,650 batches from slaughterhouse during 2012 and 2013. For all batches, transport distance (1–276 km) season and year of delivery were considered. A decrease of all defect occurrences was observed for increasing distance up to 170 km (P < 0.05). Above 170 km, however, all defects frequencies increased (P < 0.05). Season showed an effect on the incidence of defects, with an increasing of hematomas and lacerations in winter and autumn respectively (P < 0.05) and the highest percentage of veining and hemorrhages in spring (P < 0.05). Summer had the lowest incidence of defects on fresh hams. We concluded that the incidence of the examined defects and the subsequent rejection for DPO Parma ham production is lower in fresh hams transported 38–170 km during the summer.

## 1. Introduction

Pre-slaughter is known to be a critical period for pigs [1,2], because several factors, *i.e.*, inadequate transport conditions, inappropriate handling [3] and length of the journey [4], can seriously affecting animals welfare both physically and psychologically. Additionally, carcass quality can be adversely affected by inadequate pre-slaughter conditions, such as rough practices of loading and unloading and inappropriate driving style of trucks and pigs that may lead to skin wounds, hemorrhages and hematomas [2]. Increased incidence of blood-splashed and skin damage was found in pigs handled roughly or driven by electric prods during pre-slaughter [5]. These injuries represent a significant economic loss, especially if they are located on the most valuable cuts such as hams, loins and shoulders. According to Von Borrell (2005) [6], transport is considered to be a major stressor might have deleterious effects on product quality. Previous research indicates that pig welfare is affected by both long and short journeys [7]. Mota-Rojas *et al.* [8] found that the percentage of bruised carcasses increases with journey duration, while Barton Gade and Christensen [9] reported an increased risk of skin damage during short transports (2–3 h), and Gispert *et al.* [10] found higher skin damage scores in short transports (<2 h) *vs.* long transports (>2 h).

The effect of the season on pig welfare and carcass quality have been also reported [11,12]. The frequency of skin blemishes, hematomas and blood splashing was shown to increase in winter, probably caused by slips and falls due to slippery icy floor [13,14,15].

Raw hams from heavy pigs (160–170 kg live weight) reared in Northern Italy are primarily processed to DPO (Denomination Protected Origin) dry-cured hams under the control of several consortia, of which Parma Consortium is largest, branding about 10 million dry-cured hams per year (PQI 2014, PQI 2013, Prosciutto di Parma.it) [16,17]. The Parma Quality Institute (PQI) [16] inspects fresh hams destined for the DPO dry-curing process for the presence of an inappropriate physical characteristics like lean-fat ratio or physical injuries, such as hematomas, microhaemorrhages, muscle lacerations and subcutaneous veining caused by pre-slaughter handling; these lesions lead to exclusion of the cuts from the DPO Parma chain. A study of more than 100,000 fresh hams monitored by PQI in 2013 found that 4.3 % of these were rejected for DPO dry-cured ham production due to hematomas, microhaemorrhages, muscle lacerations and subcutaneous veining. Extended to the annual production of fresh hams destined to DPO Parma dry-cured ham equal to 16 million, the total number of fresh hams rejected for pre-slaughter defects could reach 689,000 with an estimated economic loss around 7.6 million euro per year for the whole Parma Consortium and Italian Food Industry.

The aim of this study was to evaluate the effects of transport distance, in connection to the slaughter season, on the incidence of defects observed on fresh hams destined for processing to DPO dry-cured ham.

## 2. Experimental Section

### 2.1. Data Sampling

This study was carried out in 2012 and 2013 on data collected from 901,990 trimmed fresh hams of heavy pigs provided in 3,650 batches supplied by 411 farmers and slaughtered over 396 days in a single plant located in Northern Italy. Each batch contained 7–151 pigs with an average of 124 animals per batch. All pigs were 9 months old with live weights of kg 171.1 ± 6.1, as required by the Parma dry-cured ham Consortium [18].

### 2.2. Pre-Slaughter Conditions and Abattoir

Transportation distances from farm to slaughterhouse ranged from 1 to 276 km and average shipping speed based on motorway proximity and road conditions was estimated at 60 km/h. The deliveries were carried out using double trailer lorries with three hydraulic deck. The lorries had natural and mechanical ventilation system, with fans placed on the left side of the trucks. Stocking density during transport was estimated ranging 141–251 kg/m^2^ and the journey duration ranged 0.25–5 h. The loading was done using the mobile ramp commonly present at the piggery (length m 6.0, width m 0.7, with solid side walls of m. 1.0 and adjustable height), while unloading at the slaughterhouse was carried out by a platform (length m 9.3, width m 2.7, solid side walls m 1.0) with adjustable height at the level of the lower deck. The vehicle was always unloaded before the rear trailer. Lairage time ranged between 30 min to 1.5 h. During this period, pigs were not mixed with unfamiliar animals. Averages and ranges of seasonal temperature and relative humidity during 2012 and 2013 in the area where pigs were transported are shown in Table 1.

**Table 1:** Temperature (°C) and Relative Humidity (%) in the slaughterhouse area during 2012 and 2013.

	**Temperature (°C)**						**Relative Humidity (%)**					
	**2012**			**2013**			**2012**			**2013**		
	Average	Range		Average	Range		Average	Range		Average	Range	
Winter	3	−2	9	4	0	8	81	58	100	85	69	100
Spring	17	11	23	16	10	21	70	44	100	73	50	100
Summer	24	17	32	23	16	30	66	39	100	70	42	100
Autumn	10	6	15	10	7	15	83	65	100	86	68	100

Pigs were driven with plastic sticks or rubber boards to resting pens, where they remained 0.5–4.0 h, with only a few batches resting overnight. After resting, pigs were showered and driven through a single chute to the stunning cage. Stunning was accomplished by electrical tongs (head only; 170 V, 1.3 A). Carcasses were then exsanguinated horizontally for 3 min and hanged vertically for 10 min before being immersed into the scalding tank. Carcasses were hot-boned and kept in the cooler (2–4 °C) overnight. The next day, hams were trimmed to the commercial shape, classified for damages and selected according to market criteria.

### 2.3. Defects Observed

Data on defects such as hematomas, lacerations, microhaemorrhages and superficial veining related to pre-slaughter practices were recorded on trimmed fresh hams. Hematomas are lumps formed by blood clots beneath the skin, typically associated with ruptured blood vessels, that can be produced by impacts against either the handling facilities or improperly used handling prods [3]. Lacerations are similar to dark hematomas distributed in the medial side of the ham and are caused by muscle tears, which may be related to slipping on wet floors during driving. Microhaemorrhages are characterized by pinpoint bleeding in the muscles due to capillary rupture and are generally attributed to rough handling, electrical stunning and vertical exsanguination [19,20,21,22]. Superficial veining is a subcutaneous venous lattice affecting the medial, or sometimes the entire, surface of the thigh. This ham defect appears several hours after slaughter, is particularly noticeable during the dressing process and is still evident at the end of the processing and seasoning [23]. The incidence and severity of the veining defect was found to be associated with the prolongation of pre-slaughter procedures (loading, transport and lairage time), with the use of CO_2_ stunning methods and with the increase of time between the separation of ham from the hot carcass and chilling from 15 min to 60 min [23]; however, the causes remain largely unexplained [19].

Trimmed hams were classified on the basis of the presence of defects, according to PQI photographic standards, by two trained slaughterhouse operators and recorded in an electronic database according to batch and producer identity. Defects were calculated as percentage per batch.

### 2.4. Statistical Analysis

Batches were classified by distance between supplier and slaughterhouse using the FASTclust procedure of Statistical Analysis System (SAS 9.3 Cary, NC, USA, 2009). Data were assigned to four clusters based on the smallest Euclidean distance from the initial seed in the cluster. The number of batches assigned to each cluster and the respective mean distances, standard deviations (SD) and minimum (min) and maximum (max) distances are reported in Table 2. Slaughter days were grouped into seasons and were monitored over two years to evaluate defect incidence change from year to year. The distribution of batches and pigs between season and year is reported in Table 3. Defect incidences approximated a Poisson distribution and were log transformed by the GLIMMIX procedure (SAS 9.3 Cary, NC, USA) prior to statistical analysis. The model included cluster, season and year and their respective interactions as fixed effects, and farm within day of slaughter as random effects. The ILINK option was used to back-transform least squares means, and differences between least squares means were evaluated by Tukey-Kramer’s test (P < 0.05).

**Table 2:** Distance and transport conditions for batches and pigs based on cluster.

Cluster (Distance)	1 (11–37 km)	2 (38–86 km)	3 (89–170 km)	4 (199–276 km)
No. of batches	1,573	990	347	740
No. of pigs	195,596	119,213	42,554	93,632
Average No. pigs/batch	124	120	123	127

Distance:				
-average (km)	21	50	121	237
-standard deviation (km)	10	9	18	12
-min (km)	1	38	89	199
-max (km)	37	86	170	276

Transport condition:				
-type of vehicle:				
single vehicle	298	246	74	112
double trailer	1,275	744	273	628
-estimated duration (h)	0.5	1–1.5	2–3	3–5
-average stocking density (kg/m²)	213	206	207	218

**Table 3:** Distribution of batches and pigs based on season and year.

Cluster (Distance)	1 (11–37 km)	2 (38–86 km)	3 (89–170 km)	4 (199–276 km)
**Season**				
Winter				
No. of batches	384	200	103	172
No. of pigs	47,765	24,581	12,881	21,759
Spring				
No. of batches	401	266	87	190
No. of pigs	49,563	31,662	10,842	24,181
Summer				
No. of batches	415	256	71	176
No. of pigs	50,496	30,659	8,458	22,608
Autumn				
No. of batches	373	268	86	202
No. of pigs	47,772	32,311	10,373	25,048
**Year**				
2012				
No. of batches	780	433	202	373
No. of pigs	96,406	52,193	26,148	46,808
2013				
No. of batches	793	557	145	367
No. of pigs	99,190	67,020	16,406	46,824

## 3. Results and Discussion

In 2012, the incidence of hematomas, lacerations and microhaemorrhages observed in trimmed hams were 4.3%, 1.7% and 1.5%, respectively. In 2013, hematomas increased to 6.2%, while lacerations and microhaemorrhages showed very similar incidences equal to 1.7% and 1.6%, respectively. During the two years, PQI, detected in the whole Parma chain (around 4200 farms and 130 slaughter plants) about twice as much the incidence of these defects [16].

The results of variance analysis and the least-squares means of defects frequency by sources of variation are reported in Table 3. Distance, season and year showed to have a significant effect on the incidence of defects under study. Significant interactions between distance × season and between distance x year were found for hemorrhages, and for hemorrages and veining, respectively. Moreover, there was a significant season x year interaction for hemorrhages, veining and laceration. Incidence of defects decreased with increasing transport distance from Cluster 1 (11–37 km) to Cluster 3 (89–170 km) (P < 0.05). The higher incidence of defects associated with short transport distances may be due to the lack of time to lie down and recovery from loading stress. Also, a reduction of standing pigs and increasing journey duration could explain this result. Lambooij (2007) showed that the percentages of slaughter pigs standing during transport decreased with journey time, which leads to a reduction in risk of slips, falls and overlaps [2]. Additionally, these results could be the consequence of truck drivers working hurriedly to accomplish all planned transports for a given day, as well as the effect of the lower quality of rural roads, which represent the major part of the route in short journeys. Nevertheless, extending of transport distance above 170 km (Cluster 4) results in an increased incidence of defects, which reaches and exceeds the frequency recorded for Cluster 1. There were no differences in hematomas and veining between clusters 4 and 1, while lacerations and hemorrhages incidences were significantly higher (P < 0.05) in Cluster 4. Thus, the incidence of defects reported herein was not proportional to the increase of transport distance. Mota-Rojas *et al.* [8] observed an increase in pig carcass bruising prolonging the journey from 8 to 23 h. Gallo *et al.* [24] identified similar trends in slaughter beef transported for 36 h. The incidence of defects on fresh hams was also influenced by season of transport (Table 4). The percentage of hematomas was greatest (P < 0.05) in winter, decreasing in spring and summer (P < 0.05) and reaching the lowest incidence in autumn (P < 0.05). There are no differences between winter, spring and summer for lacerations, while the incidence of this defect was greater (P < 0.05) in autumn. Gosàlvez *et al.* [15] reported a higher incidence of bruised carcasses in pigs transported in winter and in autumn. Scheeren *et al.* [13] hypothesize that the greater proportion of skin damage in winter is linked to the tendency of pigs to stand during transportation to avoid making contact with the cold floor and walls of the trailer. Dalla Costa *et al.* [25] found that pigs were more difficult to handle in winter and need more coercion, which led to more handling-induced bruises. The higher incidence of lacerations observed in autumn could be related to the increase of live weight in this season, due to a restarting of feed intake for the decrease of temperature with respect to the summer. The average live weights recorded by the slaughterhouse during 2012 and 2013 confirms that in autumn there was an increase of more than one kg with respect to the summer (169.1 *vs.* 170.6 kg in 2012 and 169.4 *vs.* 170.9 kg in 2013). This seasonal increase of live weight of heavy pigs was also shown by the national slaughter statistics of heavy pigs [26].

The incidence of hemorrhages was greater (P < 0.05) in spring and in autumn while the veining defect was greater in spring. These results cannot easily be attributed to differences in seasonal conditions. The lowest occurrences of lacerations and hemorrhages were found in summer. Warmer temperatures recorded during this season probably lead to a more careful handling in order to reduce the risk of transport mortality [15,25,27]. It is well known that high environmental temperature is a risk factor for transport mortality [28,29].

A significant source of variation in the incidence of raw ham defects was found in studies over several years. The higher incidence of all defects was found in 2013, especially for hematomas and veining. Crisis-invested DPO dry-cured ham production became more important in 2013 with respect to the previous year. It is probably that the reduction of employees for reducing costs lead to an overworking of staff involved in transport and in pre-slaughter handling with a consequent reduction of its quality. The detrimental effect to animal welfare due to overworking of employees involved in animal handling was highlighted by Grandin [30].

**Table 4:** Results of variance analysis and least-squares means of defects incidence by sources of variation.

**Sources of variation**	Hematomas (%)	Lacerations (%)	Hemorrhages (%)	Veining (%)
	P / Least squares mean	P / Least squares mean	P / Least squares mean	P / Least squares mean
**Distance**	**0.0079**	**<0.0001**	**<0.0001**	**<0.0001**
1 (11–37 km)	4.57 a	1.48 b	1.32 b	11.04 a
2 (38–86 km)	4.44 ab	1.31 c	1.34 b	11.14 a
3 (89–170 km)	4.08 b	1.30 bc	1.08 c	9.24 b
4 (199–276 km)	4.78 a	1.91 a	1.77 a	11.50 a
Pooled SEM(*)	0.11	0.06	0.05	0.22
				
**Season**	**<0.0001**	**<0.0001**	**<0.0001**	**<0.0001**
Winter	6.53 a	1.32 b	1.19 b	12.05 b
Spring	5.88 b	1.33 b	1.74 a	15.23 a
Summer	3.58 c	1.28 b	0.96 c	8.70 c
Autumn	2.87 d	2.13 a	1.71 a	8.18 c
Pooled SEM(*)	0.12	0.06	0.05	0.24
				
**Year**	**<0.0001**	**0.0359**	**<0.0001**	**<0.0001**
2012	3.70 b	1.42 b	1.24 b	9.73 b
2013	5.37 a	1.55 a	1.48 a	11.74 a
Pooled SEM(*)	0.08	0.04	0.04	0.04
				
**Distance × season**	**0.7421**	**0.3412**	**0.0352**	**0.5894**
**Distance × year**	**0.1526**	**0.7083**	**0.0137**	**0.0007**
**Season × year**	**0.1926**	**0.0056**	**<0.0001**	**<0.0001**

(*) SEM: Standard Error of Mean; Means in the same column without the same letter differ significantly (P < 0.05).

Interaction between distance and season (Table 5) affected the incidence of hemorrhages (P < 0.05). The defect increased markedly when distance increased above 170 km in spring and in autumn (P < 0.05). Differences between seasons were observed for less than 37 km distance (Cluster 1), with summer showing the lowest incidence of hemorrhages (P < 0.05). Above this distance, the lowest incidences of the defect were found in summer and in winter (P < 0.05).

**Table 5:** Effect of interaction between distance and season on the incidence of hemorrhages.

	Cluster (Distance)				Pooled SEM
	1 (11–37 km)	2 (38–86 km)	3 (89–170 km)	4 (199–276 km)	
**Hemorrhages (%)**					
Winter	1.36 ab x	1.14 ab y	0.87 b y	1.49 a y	0.0931
Spring	1.55 b x	1.71 b x	1.58 b x	2.16 a x	0.1096
Summer	0.98 ab y	1.00 ab y	0.65 b y	1.33 a y	0.0912
Autumn	1.48 b x	1.67 b x	1.52 b x	2.26 a x	0.1527

Least square means within a row with different letters (a–b) differ (P < 0.05); Least square means within a column with different letters (x–y) differ (P < 0.05).

Interaction between distance and year (Table 6) affected the frequencies of hemorrhages and veining. They increased both in 2012 and 2013 when distance increased above 170 km. The lowest incidences of these defects (P < 0.05) were found for cluster 3 (89–170 km) in 2012 only.

**Table 6:** Effect of interaction between distance and year on the incidence of hemorrhages and veining defects.

	Cluster (Distance)				Pooled SEM
	1 (11–37 km)	2 (38–86 km)	3 (89–170 km)	4 (199–276 km)	
**Hemorrhages (%)**					
2012	1.32 a	1.27 a	0.91 b	1.57 a x	0.0688
2013	1.33 b	1.42 b	1.28 b	1.99 a y	0.0796

**Veining (%)**					
2012	10.39 a x	10.63 a	7.63 b x	10.64 a x	0.2838
2013	11.73 y	11.68	11.17 y	12.42 y	0.3366

Least square means within a row with different letters (a–b) differ (P < 0.05); Least square means within a column with different letters (x–y) differ (P < 0.05).

Interaction between season and year (Table 7) affected the incidences of lacerations, hemorrhages and veining. No differences between years were observed for lacerations but these differences appeared for hemorrhages in summer and autumn and for veining in spring, summer and autumn. These results confirmed the effects of the season on the incidences of raw ham defects. With regards to the influence of the year, as stated before, its effect appears related to a reduction of the quality of animal handling.

**Table 7:** Effect of interaction between season and year on the incidence of lacerations, hemorrhages and veining defects.

	Season				Pooled SEM
	Winter	Spring	Summer	Autumn	
**Lacerations (%)**					
2012	1.19 b	1.31 b	1.16 b	2.23 a	0.0824
2013	1.47 b	1.35 b	1.41 b	2.04 a	0.0873

**Hemorrhages (%)**					
2012	1.28 b	1.71 a	0.71 c x	1.53 b x	0.0711
2013	1.11 b	1.76 a	1.30 b y	1.90 a y	0.0802

**Veining (%)**					
2012	11.40 b	17.55 a x	6.72 c x	6.60 c x	0.3040
2013	12.73 a	13.06 a y	11.27 ab y	10.14 b y	0.3369

Least square means within a row with different letters (a–c) differ (P < 0.05); Least square means within a column with different letters (x–y) differ (P < 0.05).

## 4. Conclusions

The study has examined the effects of travel distances from 1 to 276 km, showing that both short and long journeys may have adverse effects on fresh ham defects. The higher incidence of defects associated with short transport distances may be due to the lack of time to lie down and recovery from loading stress, but probably also to hauliers working quickly to accomplish all planned transports for a given day. Additionally, these results could be related to the lower quality of rural roads, which represent the major part of the route in short journeys. Extending the transport distance above 170 km results in an increased incidence of raw ham defects; the incidence of defects reported herein was not proportional to the increase of transport distance This research highlights the need for Italian pig industry to improve pre-slaughter handling in order to increase the quality of raw hams for DPO production, reducing the economic losses related to inadequate practices before slaughter and increasing the welfare of animals to make it as good as possible. However, additional research is necessary to understand seasonal influences on frequencies of defects on the fresh hams destined for the DPO process.
